# Ethics in the twenty first century otolaryngology^☆^

**DOI:** 10.1016/j.bjorl.2017.01.001

**Published:** 2017-01-19

**Authors:** Charles W. Cummings

**Affiliations:** Johns Hopkins University, Baltimore, USA

In the United States, the climate of the practice of Medicine is in transition and under siege by policy revisionists and governmental/political opportunists. Regulatory and legislative hubris has enshrouded the medical arena through formulaic dictums and onerous regulations oft times constructed by individuals who have no clinical experience or knowledge. The art of Medicine is being dismantled by emphasis on outcomes based solely on statistical evidence, with no concern for the human variables. Objective analysis is not inherently bad provided the personal and social factors are properly emphasized in the analysis. Freedom to make clinical decisions MUST be preserved for optimal care.

This phenomenon exerts undue pressure on the clinician leading to professional frustration and anemia of job satisfaction. Also, the environment which enables the best of care is further challenged and compromised.

Never the less, we, as physicians, must continue to acknowledge our calling and respond to the gifts of our profession. It seems appropriate to review the commitments that we have made to ourselves and our patients. Over the years, I have created a wish list concerning the way I want to conduct myself to satisfy my professional goals. What follows are those wishes. I strongly recommend that all practicing physicians consider such a list, and review it occasionally.

1. I wish for the judgement to know what is best for my patients and to weigh the merits of interventions; surgically, medically, and radiotherapeutically.
2. I wish that I can master the complexities of the practice of medicine and be worthwhile as a physician.
3. I wish for the honesty and integrity to objectively assess my competencies and fallibilities.
4. I wish for the intellectual drive to maintain currency of knowledge.
5. I wish for the wisdom to assess and maintain appropriate costs for medical care.
6. I wish for the integrity to avoid marketing my skills and accomplishments unrealistically and erroneously.
7. I wish for the compassion that allows me to separate my personal problems from the needs of the patients that I serve.
8. I wish for the courage to challenge the wisdom of those that create policies and regulations that fail to consider the full dimension and scope of medical care.

Please preserve the art of Medicine. We must steward our wonderful calling.

## Conflicts of interest

The author declares no conflicts of interest.

